# Bone Marrow Lesions and Magnetic Resonance Imaging–Detected Structural Abnormalities in Patients With Midfoot Pain and Osteoarthritis: A Cross‐Sectional Study

**DOI:** 10.1002/acr.24955

**Published:** 2022-12-02

**Authors:** John B. Arnold, Jill Halstead, Carmen Martín‐Hervás, Andrew J. Grainger, Anne‐Maree Keenan, Catherine L. Hill, Philip G. Conaghan, Dennis McGonagle, Anthony C. Redmond

**Affiliations:** ^1^ University of Leeds and NIHR Leeds Biomedical Research Centre, Leeds, UK, and IIMPACT in Health, Allied Health and Human Performance, University of South Australia Adelaide South Australia Australia; ^2^ University of Leeds, NIHR Leeds Biomedical Research Centre, and Leeds Community Healthcare NHS Trust Leeds UK; ^3^ La Paz University Hospital, Universidad Autónoma de Madrid and Biomedical Research Networking Centre on Bioengineering, Biomaterials and Nanomedicine, Ciber‐BBN Madrid Spain; ^4^ University of Leeds and NIHR Leeds Biomedical Research Centre, Leeds, and Cambridge University Hospitals NHS Foundation Trust and University of Cambridge Cambridge UK; ^5^ University of Leeds and NIHR Leeds Biomedical Research Centre Leeds UK; ^6^ The Queen Elizabeth Hospital and The University of Adelaide Adelaide South Australia Australia

## Abstract

**Objective:**

To compare magnetic resonance imaging (MRI)–detected structural abnormalities in patients with symptomatic midfoot osteoarthritis (OA), patients with persistent midfoot pain, and asymptomatic controls, and to explore the association between MRI features, pain, and foot‐related disability.

**Methods:**

One hundred seven adults consisting of 50 patients with symptomatic and radiographically confirmed midfoot OA, 22 adults with persistent midfoot pain but absence of radiographic OA, and 35 asymptomatic adults underwent 3T MRI of the midfoot and clinical assessment. MRIs were read for the presence and severity of abnormalities (bone marrow lesions [BMLs], subchondral cysts, osteophytes, joint space narrowing [JSN], effusion‐synovitis, tenosynovitis, and enthesopathy) using the Foot Osteoarthritis MRI Score. Pain and foot‐related disability were assessed with the Manchester Foot Pain and Disability Index.

**Results:**

The severity sum score of BMLs in the midfoot was greater in patients with midfoot pain and no signs of OA on radiography compared to controls (*P* = 0.007), with a pattern of involvement in the cuneiform–metatarsal joints similar to that in patients with midfoot OA. In univariable models, BMLs (ρ = 0.307), JSN (ρ = 0.423), and subchondral cysts (ρ = 0.302) were positively associated with pain (*P* < 0.01). In multivariable models, MRI abnormalities were not associated with pain and disability when adjusted for covariates.

**Conclusion:**

In individuals with persistent midfoot pain but no signs of OA on radiography, MRI findings suggested an underrecognized prevalence of OA, particularly in the second and third cuneiform–metatarsal joints, where BML patterns were consistent with previously recognized sites of elevated mechanical loading. Joint abnormalities were not strongly associated with pain or foot‐related disability.

## INTRODUCTION

Foot osteoarthritis (OA) is increasingly being recognized as an important contributor to the overall burden of OA (1), affecting 1 in 6 adults >50 years of age (2). Midfoot OA is the most disabling location of foot OA (3) and is associated with persistent pain and difficulty walking and carrying out of day‐to‐day activities (3, 4). Treatment of midfoot OA consists of pharmacologic pain management and strategies for joint protection to offload the midfoot, such as supportive footwear and foot orthoses (5). Unfortunately, clinical effects are modest and symptoms are often persistent (6). The lack of effective treatments for midfoot OA is, in part, due to a lack of understanding about sources and mechanisms of midfoot pain suggesting potential treatment targets.

**SIGNIFICANCE & INNOVATIONS**

- This is the first detailed investigation of magnetic resonance imaging (MRI)–detected abnormalities in patients with symptomatic radiographic midfoot osteoarthritis (OA) and patients with persistent midfoot pain but an absence of radiographic changes.
- MRI findings identified a previously unrecognized prevalence of bone marrow lesions (BMLs) in patients with midfoot pain but no signs of OA on radiography.
- The pattern of BML involvement in the cuneiform–metatarsal joints was similar to that in patients with midfoot OA and consistent with previously recognized sites of elevated mechanical loading.
- BMLs and cysts were positively but weakly associated with foot pain and not in multivariable models after adjustment for covariates.

Plain radiography has been the mainstay for diagnosing midfoot OA; however, it is well recognized that OA is a disease of the whole joint involving both bone and soft‐tissue structures (7). Insights from other joint sites such as the hand (8), knee (9), and hip (10) suggest that features not visible on radiography and potential sources of nociceptive input such as bone marrow lesions (BMLs) and synovitis are important drivers of symptoms (11).

Magnetic resonance imaging (MRI) has greatly expanded our understanding of OA and the search for modifiable treatment targets (12). The multiplanar and multi‐tissue visualization afforded by MRI allows assessment of bone and soft tissue pathologies, and structural changes can be visualized before they become apparent on radiography. Evidence from studies of patients with persistent knee pain suggest that preradiographic, MRI‐detected abnormalities precede the development of knee symptoms and knee OA, highlighting the potential value of early identification of structural pathology (13, 14). In the midfoot, however, no studies have investigated using MRI which joint features may be associated with pain and whether structural abnormalities are associated with foot pain and disability.

We hypothesized that patients with midfoot OA and those with persistent midfoot pain without radiographic OA would exhibit a greater prevalence of MRI structural abnormalities compared to asymptomatic controls. The first aim of this study was, therefore, to compare assessed structural abnormalities in patients with symptomatic midfoot OA, those with persistent midfoot pain but confirmed absence of radiographic OA, and asymptomatic adults. The second aim was to explore the association between MRI features and foot pain and disability.

## PATIENTS AND METHODS

### Study design and recruitment

This was a cross‐sectional study involving participants with 1) symptomatic and radiographically confirmed midfoot OA, 2) persistent midfoot pain but confirmed absence of radiographic OA, and 3) asymptomatic adults with no foot symptoms (as a comparative control). Participants were recruited from the community via advertisements, general practitioners, and health clinics. Patients were recruited during 2 time periods, from June 2009 to February 2011, and December 2017 to August 2018. Controls were recruited from August to December 2018. Ethical approval was obtained from the Leeds East and West Research Ethics Committee (09/H1305/10 and 17/YH/0261). All participants provided written informed consent prior to their involvement.

### Participants

Participants with symptomatic midfoot OA were >40 years of age and had pain in the midfoot for >3 months with an average pain severity in the past week of ≥3 of 10 on an 11‐point numerical rating scale (NRS), occurring with or worsening following weight‐bearing activities. The location of midfoot pain was confirmed on a foot manikin (15, 16), with midfoot pain defined as per the manikin templates previously published (15). Pain in specific joints was determined via pain on palpation of the talonavicular, navicular–cuneiform, or cuneiform–metatarsal joints. Additionally, weight‐bearing dorsoplantar and lateral radiographs were used to confirm and grade OA in the talonavicular, navicular–first cuneiform, cuneiform–first metatarsal, or cuneiform–second metatarsal joint by an experienced specialist musculoskeletal radiologist (AJG) using the La Trobe Foot Atlas (17). A joint was considered to have OA with a score of ≥2 for osteophytes or joint space narrowing (JSN) on either the dorsoplantar or lateral views (17). Intrarater reliability for scoring was excellent, as previously reported (κ = 0.92 [95% confidence interval (95% CI) 0.90–0.95]) (18).

Participants with persistent midfoot pain also had localized pain in the midfoot region for >3 months (nontraumatic in origin, occurring with or worsening following weight‐bearing activity) but had no clear clinical signs of midfoot OA (including observed/palpable osteophytes, crepitus, and reduced joint motion). Standard anteroposterior and oblique radiographs also demonstrated no features of midfoot OA (osteophytes or JSN).

Exclusion criteria for the groups with symptomatic midfoot OA or persistent midfoot pain were >30 minutes of early morning stiffness in the feet, inflammatory arthritis, muscle or connective tissue disease, neurologic conditions (including diabetes mellitus), peripheral arterial disease, glucocorticoid injection to the foot in the past 6 months, stress fracture or history of foot surgery, or contraindications to MRI. Concurrent knee or hip pain was permitted if the pain intensity was <2 of 10 in the past week on an NRS.

Asymptomatic controls were >40 years of age and had no foot or lower limb joint pain in the past 7 days, confirmed using an 11‐point NRS for foot pain and a body manikin. Additional exclusion criteria for controls included presence of radiographic OA (osteophytes or JSN >1) in any of the midfoot joints (talonavicular, navicular–first cuneiform, cuneiform–first metatarsal, or cuneiform–second metatarsal joint), inflammatory arthritis, muscle or connective tissue disease, neurologic conditions, stress fracture, or lower limb bone or joint surgery in the past 12 months.

### Foot MRI

The midfoot joints of each participant's most painful foot (or right foot for controls) were examined using a Magnetom Verio 3T MR system (Siemens). All images were acquired using an 8‐channel foot and ankle coil, with the foot placed perpendicular to the leg and magnetic field. The following protocol and parameters were employed: T2‐weighted fat‐saturated sequence repetition time (TR) 3,000–3,600 msec, echo time (TE) 69 msec, flip angle 155–160°, an echo train length of 8, 2‐mm slices and a 0.4‐mm interslice gap, matrix 256 × 256, and field of view (FOV) 150 × 150 mm in 3 planes; STIR sequence TR 4,500 msec, TE 31 msec, number of excitations 2, time to inversion 200 msec, flip angle 150°, an echo train length of 11, 3‐mm slices and a 0.6‐mm interslice gap, matrix 320 × 256, and FOV 150 × 150 mm in 3 planes; and T1‐weighted high‐resolution spin‐echo TR 700 msec, TE 10 msec, flip angle 90°, 1.2‐mm slices and a 1.32‐mm interslice gap, matrix 512 × 512, and FOV 512 × 512 mm in the sagittal and coronal plane.

### Semiquantitative imaging scoring

Scans were scored using Osirix, version 5.6 (Osirix). A single musculoskeletal specialist radiologist with 15 years of experience (CMH) read and scored the images for the midfoot joints and bones using the Foot Osteoarthritis MRI Score (FOAMRIS) (19). The FOAMRIS includes dichotomous (0–1) or criterion‐based ranking (0–3) scores for JSN, osteophytes, BMLs, cysts (0–1), joint effusion‐synovitis (0–1), and soft‐tissue features including tenosynovitis and enthesopathy (0–1) (tibialis anterior, extensor hallucis longus, extensor digitorum longus, fibularis brevis, fibularis longus, tibialis posterior, flexor hallucis longus, and flexor digitorum longus). The number of joints affected in the midfoot and at the joint level (score of 1 for features scored 0–1, or ≥2 for features scored 0–3) were calculated, as well as severity sum scores (tallied scores in the combined midfoot joints for each feature). MRI scans were scored blind to clinical and pain status. The FOAMRIS displays good intrareader reliability and fair interreader reliability when assessing total feature scores (19), with the scoring system yet to undergo validation with other imaging modalities or clinical outcomes.

### Assessment of foot pain and disability

Pain severity in the past 24 hours was documented with a 100‐mm visual analog scale. Pain and foot‐related disability were assessed using the Manchester Foot Pain and Disability Index (MFPDI) (20). The MFPDI is a 19‐item questionnaire that assesses foot pain (5 items), disability (10 items), appearance (2 items), and impact on work or leisure (2 items). Each item is scored from 0 (none of the time) to 2 (on most/every day). Pain subscale scores range from 0 to 10, and function scores from 0 to 20, with higher scores indicating more pain or worse foot‐related disability. The MFPDI has previously been used in patients with midfoot OA (3, 6, 21) and displays good construct validity and internal consistency (20). Prior to analysis, raw scores were converted to Rasch‐transformed interval‐level scores. As the work and leisure questions are not applicable to all individuals, the results for this subscale are not reported.

### Statistical analysis

Descriptive statistics were generated for demographic and clinical characteristics. Medians and interquartile ranges were used to describe the number of affected joints and severity sum scores for patients with midfoot OA, patients with midfoot pain only, and asymptomatic controls. Differences in affected joints and sum scores between groups were compared using generalized linear models (normal distribution family and identity link function) and adjusted for age, sex, and body mass index (BMI). Optimal model fit for this family and link function was verified using Akaike's information criterion. At the joint level, the proportion of joints classified as affected were compared between groups using chi‐square tests. Affected joints were defined as a score of 1 for features scored dichotomous (0–1), or ≥2 for those features scored on a severity scale of 0 to 3. Univariate associations between severity sum scores for each feature, age, and BMI with MFPDI pain and disability subscales was assessed with Spearman's rank correlation coefficients. Correlations that were statistically significant (*P* value less than 0.05) were included in multivariable linear regression models as independent variables with MFPDI pain and disability scores (dependent variables) with covariates of age, sex, and BMI. Results are presented as unstandardized beta coefficients with 95% CIs. The level of significance was set at α = 0.05.

## RESULTS

### Descriptive and clinical characteristics

In total, 367 potential participants with symptoms were screened for eligibility, with 77 (21%) meeting the criteria for the midfoot OA (n = 50) or persistent midfoot pain group (n = 22). Sixty patients were recruited from general practitioners or health clinics, and 17 responded to community advertisements. Seventy‐six potential asymptomatic control participants were screened, with 35 meeting the criteria (Table 1). Patients with midfoot OA and asymptomatic controls were similar in age, with the radiographically negative midfoot pain group being younger. Groups were comparable for sex and BMI. The midfoot OA group reported moderate levels of pain over the past 24 hours (mean ± SD 36 ± 21 on 0–100 visual analog scale), which was similar to those with midfoot pain but no radiographic OA (mean ± SD 37 ± 19). Foot pain assessed with the MFPDI revealed more frequent and troubling symptoms in patients with midfoot OA (mean ± SD 6.0 ± 1.3) compared to those with midfoot pain only (mean ± SD 3.1 ± 1.6), but foot‐related disability was similar (mean ± SD 9.2 ± 2.3 versus 8.6 ± 3). Patients with midfoot OA most commonly had radiographic OA in the cuneiform–second metatarsal joint (74%), followed by the navicular–first cuneiform joint (40%), cuneiform–first metatarsal joint (36%), and the talonavicular joint (20%) (Table 1).

**Table 1:** Descriptive characteristics of participants with symptomatic midfoot osteoarthritis (OA), participants with persistent midfoot pain, and asymptomatic controls*

Variable	Asymptomatic adults (n = 35)	Midfoot pain (n = 22)	Symptomatic midfoot OA (n = 50)
Demographic characteristics			
Age, years	63.5 ± 11.9	44.7 ± 16.8	61.8 ± 11.3
Sex, % female	68	63	76
BMI, kg/m^2^	27.2 ± 5.4	28.8 ± 4.5	29.2 ± 4.8
Foot pain and function			
MFPDI Rasch pain score†	–	3.1 ± 1.6	6.0 ± 1.3
MFPDI Rasch function score†	–	9.2 ± 2.5	8.6 ± 3.0
MFPDI Rasch appearance score†	–	1.8 ± 1.3	1.5 ± 1.3
Foot pain severity score, last 24 hours‡	0 ± 0	37.4 ± 19.9	36.7 ± 21
Joint‐specific radiographic OA§			
Talonavicular joint, no. (%)	–	–	10 (20)
Navicular–first cuneiform joint, no. (%)	–	–	20 (40)
First cuneiform–metatarsal joint, no. (%)	–	–	18 (36)
Second cuneiform–metatarsal joint, no. (%)	–	–	37 (74)

* Values are the mean ± SD unless indicated otherwise. BMI = body mass index; MFPDI = Manchester Foot Pain and Disability Index.

† Higher value indicates more foot pain, foot‐related disability, or concern with foot appearance.

‡ Pain severity measured on 0–100 mm visual analog scale, with higher scores indicating worse pain.

§ Joint‐specific OA does not tally to 100%, as ≥1 midfoot joint may have OA.

### Prevalence of MRI‐detected abnormalities and comparisons across clinical groups

The total severity sum scores and number of joints/bones with each MRI abnormality in each group is presented in Table 2. Bone marrow lesion sum scores were statistically significantly different between all groups (*P* < 0.05). Patients with midfoot pain only had greater scores compared to asymptomatic controls (*P* = 0.007) and lesser scores compared to the midfoot OA group (*P* = 0.009). Patients with midfoot OA had greater severity sum scores compared to those with midfoot pain only and asymptomatic controls for BMLs (6.0 versus 2.5 and 1.0, respectively) and JSN (5.0 versus 1.0 and 4.5, respectively). Sum scores for osteophytes (5 versus 4) and subchondral cysts (2 versus 0) were greater in patients with midfoot OA compared to controls and patients with midfoot pain, respectively. The prevalence of tenosynovitis as determined by increased tendon‐associated joint fluid was greater in patients with midfoot pain compared to asymptomatic controls (median 3.5 tendons affected versus 2.0; *P* < 0.05). Effusion‐synovitis was more common in patients with midfoot pain and asymptomatic controls compared to midfoot OA, although this feature was frequently present (7–9 joints of 10 across groups).

**Table 2:** Total sum scores for each abnormality on magnetic resonance imaging (MRI) and number of midfoot joints (tarsometatarsal, navicular–cuneiform, talonavicular, and calcaneocuboid joints) and midfoot bones with each MRI abnormality*

MRI feature	Asymptomatic adults (n = 35)	Midfoot pain (n = 22)	Midfoot OA (n = 50)
Total sum score for MRI abnormality			
Osteophytes (range 0–30)	4 (3)	4.5 (3)	5 (3)†
Joint space narrowing (range 0–30)	4.5 (4)	1 (2)	5 (4)‡
Bone marrow lesions (range 0–30)	1 (2)	2.5 (3)†	6 (5)‡
Subchondral cysts (range 0–10)	1 (1)	0 (1)	2 (2)†
Effusion‐synovitis (range 0–10)	9 (4)	8 (3)§	7 (4)
Tenosynovitis (no. of tendons, range 0–8)	2 (2)	3.5 (2)†	2.5 (2)
Enthesopathy (no. of entheses, range 0–14)	0 (2)	1 (2)	1 (1)
No. of joints with MRI abnormality			
Osteophytes (range 0–10)¶	0 (0)	1 (1)	1 (1)
Joint space narrowing (range 0–10)¶	0 (1)	0 (0)	1 (2)
Bone marrow lesions (range 0–10)¶	0 (0)	0 (1)	1 (2)
Subchondral cysts (range 0–10)¶	1 (1)	0 (1)	2 (2)
Effusion‐synovitis (range 0–10)#	9 (4)	8 (3)	7 (4)
Tenosynovitis (no. of tendons, range 0–8)¶	2 (2)	3.5 (2)	2.5 (2)
Enthesopathy (no. of entheses, range 0–14)#	0 (2)	1 (2)	1 (1)

* Values are the median (interquartile range) unless indicated otherwise. OA = osteoarthritis.

† Statistically significant compared to control with adjustment for age, sex, and body mass index (BMI) (*P* < 0.05).

‡ Statistically significant compared to control with adjustment for age, sex, and BMI (*P* < 0.05). Statistically significant compared to midfoot pain group with adjustment for age, sex, and BMI (*P* < 0.05).

§ Statistically significant compared to midfoot OA group with adjustment for age, sex, and BMI (*P* < 0.05).

¶ Scored as present if ≥2.

# Scored as present if >0.

**Figure 1:**
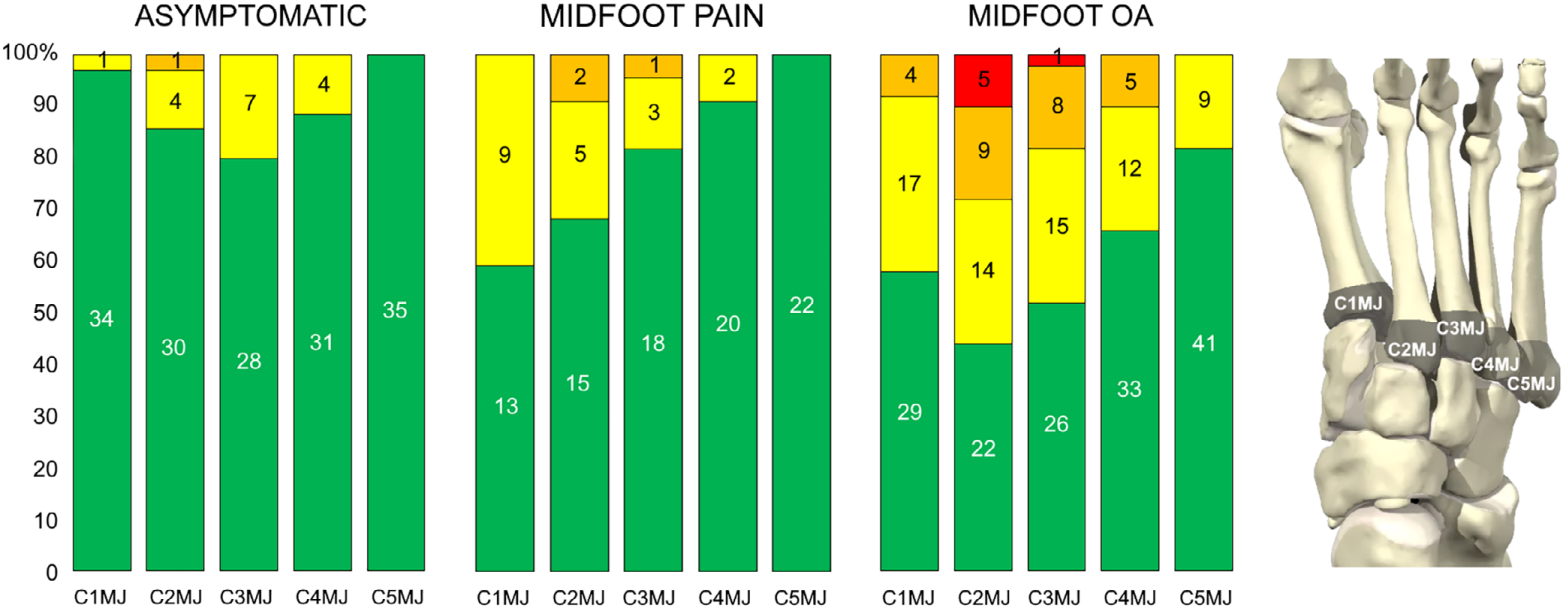
Presence and severity of bone marrow lesions in the first to fifth metatarsal joint (MJ) bases for asymptomatic adults, patients with midfoot pain only, and patients with midfoot osteoarthritis (OA). Severity of bone marrow lesion in each bone was scored ranging from 0 to 3 according to the proportion of the metatarsal base with an abnormal signal: 0 (green) = none; 1 (yellow) = 1–33%; 2 (orange) = 34–66%; and 3 (red) = 67–100%. C = cuneiform.

Joint‐level comparisons indicated that bone marrow lesions were more prevalent in the second and third metatarsal bases and medial and lateral cuneiforms in patients with midfoot OA compared to the other groups (*P* < 0.05) (Figure 1). The location and pattern of involvement, however, was similar in the 2 symptomatic groups, favoring the first to third metatarsal bases and medial, intermediate, and lateral cuneiforms (Figure 1). Full results for joint‐level comparisons are provided in Supplementary Tables 1–4 and Supplementary Figure 1, available on the *Arthritis Care & Research* website at http://onlinelibrary.wiley.com/doi/10.1002/acr.24955.

**Figure 2:**
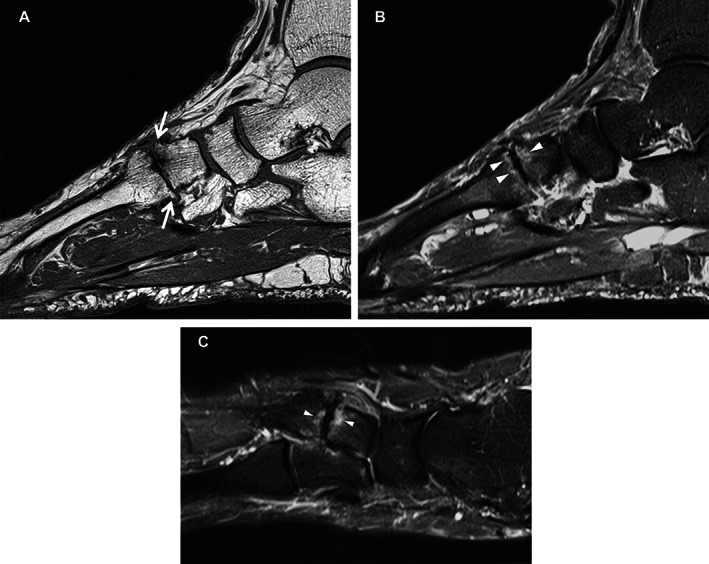
Study patient with midfoot pain. Sagittal T1 (**A**), sagittal STIR (**B**), and long axis STIR (**C**) imaging demonstrate subchondral edema on both sides of the second tarsometatarsal joint (**arrowheads**) along with osteophyte formation (**arrows**).

**Figure 3:**
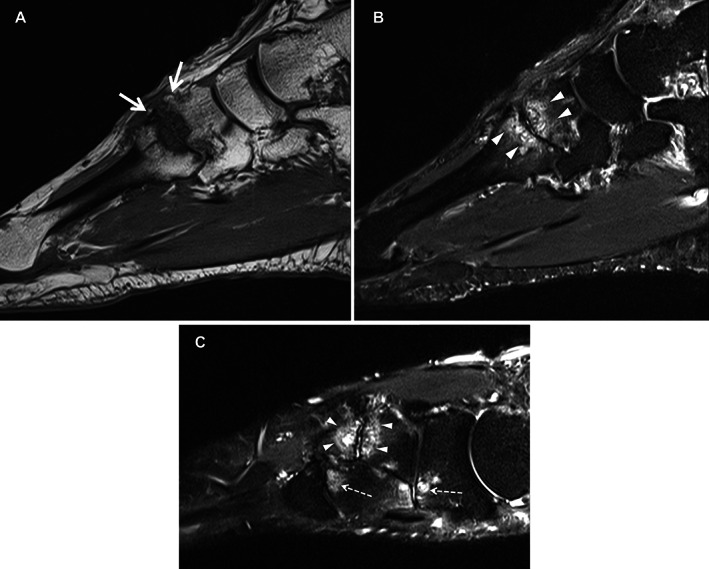
Study patient with symptomatic midfoot osteoarthritis. Sagittal T1 (**A**), sagittal STIR (**B**), and long axis STIR (**C**) imaging show subchondral edema and cyst formation on both sides of the second tarsometatarsal joint (**arrowheads**) along with osteophyte formation (**arrows**). Full‐thickness cartilage loss is also evident. There is also evidence of subchondral edema at the navicular–lateral cuneiform and third tarsometatarsal joints (**broken arrows**).

Abnormalities for JSN were more prevalent in patients with midfoot OA at the second and third cuneiform–metatarsal joint, and osteophytes at the fourth cuneiform–metatarsal joint. The prevalence of JSN and osteophytes in any midfoot joint on MRIs in patients with midfoot pain but no radiographic OA (i.e., MRI‐detected abnormalities) was 14% and 23%, respectively. Cysts were more prevalent in the navicular–intermediate cuneiform joints and the cuneiform–third metatarsal joint in patients with midfoot OA. Tenosynovitis of the fibularis longus tendon was more frequent in patients with midfoot OA, and signal abnormality of the tibialis posterior tendon at the navicular groove (functional enthesis [22]) was more common in patients with midfoot pain compared to asymptomatic adults and patients with midfoot OA. Example MR findings in the second tarsometatarsal joint for patients with midfoot pain only and symptomatic midfoot OA are shown in Figures 2 and 3.

### Associations between MRI‐detected abnormalities, foot pain, and foot‐related disability

In patients with midfoot OA or midfoot pain only, foot pain was positively correlated with BMLs (ρ = 0.307, *P* = 0.009), JSN (ρ = 0.423, *P* < 0.001), and subchondral cysts (ρ = 0.302, *P* = 0.010) (Table 3). Enthesopathy was positively associated with foot‐related disability (ρ = 0.237, *P* = 0.046). Age was positively correlated with JSN (ρ = 0.584), BMLs (ρ = 0.432), osteophytes (ρ = 0.466), and cysts (ρ = 0.520, *P* < 0.001 for all), and BMI was negatively associated with tenosynovitis (ρ = –0.299, *P* = 0.011). In multivariable models, BMLs, JSN, and cysts were not independently associated with foot pain in a model that included age, sex, and BMI. Enthesopathy was not independently associated with foot‐related disability after adjustment for covariates (see Supplementary Table 5, available on the *Arthritis Care & Research* website at http://onlinelibrary.wiley.com/doi/10.1002/acr.24955). Age was the most influential covariate in attenuating the relationship between joint abnormalities and pain, and BMI most strongly attenuated the relationship for foot‐related disability (data not shown).

**Table 3:** Associations between sum scores for abnormalities detected on magnetic resonance imaging (MRI) with foot pain, function, and physical characteristics in patients with persistent midfoot pain and midfoot osteoarthritis*

MRI feature	MFPDI pain		MFPDI function		Age		BMI	
	ρ	*P*	ρ	*P*	ρ	*P*	ρ	*P*
Joint space narrowing	0.423	<0.001†	0.020	0.867	0.584	<0.001†	0.064	0.592
Bone marrow lesions	0.307	0.009†	–0.065	0.590	0.432	<0.001†	–0.009	0.939
Osteophytes	0.226	0.062	0.119	0.330	0.466	<0.001†	0.002	0.984
Cysts	0.302	0.010†	0.064	0.594	0.520	<0.001†	0.099	0.409
Effusion‐synovitis	0.025	0.834	0.083	0.496	0.187	0.118	–0.079	0.513
Tenosynovitis	–0.088	0.465	0.088	0.467	0.073	0.542	–0.299	0.011†
Enthesopathy	–0.067	0.580	0.237	0.046†	–0.100	0.402	0.054	0.654

* BMI = body mass index; MFPDI = Manchester Foot Pain and Disability Index.

† Significant (*P* < 0.05).

## DISCUSSION

Midfoot OA is the most disabling form of foot OA, yet little is known about the type and extent of MRI‐detected joint changes, including in those patients with persistent midfoot pain but no radiographic evidence of OA. Studies investigating primary midfoot OA are mostly absent, as radiographs and computed tomography imaging modalities are favored in surgical studies that are common in the OA literature (23, 24). In this study, we identified that BMLs appear to be a feature that can distinguish patients with persistent midfoot pain from asymptomatic controls. Given the spatial association between BMLs and cartilage loss (25), these findings suggest an underrecognized prevalence of nonradiographic detected OA in patients with persistent midfoot pain. The location and pattern of involvement, primarily in the second and third cuneiform–metatarsal joints, were similar to those in patients with midfoot OA and consistent with recognized sites of elevated mechanical loading. Severity scores for JSN, BMLs, cysts, and enthesopathy were not strongly associated with foot pain and foot‐related disability and were not independently associated with symptoms after adjustment for covariates.

BMLs were identified as a feature that distinguished patients with persistent midfoot pain and midfoot OA from asymptomatic controls. As low‐grade BMLs on fluid‐sensitive MR sequences can also be found in asymptomatic individuals (26, 27), our study included a control group to help define the clinical significance of any BML involvement. The pattern of BML involvement favored the medial midfoot, in particular the medial and lateral cuneiforms, and second and third metatarsal bases. BMLs represent areas of microdamage and remodeling (28), in part precipitated by mechanical overload (29). This distribution of BMLs found in this study is consistent with the loading conditions across the cuneiform–metatarsal joints, with the highest contact forces in the second and third tarsometatarsal joint, followed by the first and fourth/fifth joints (30). The pattern of BML involvement was also consistent between those with midfoot pain and nonradiographic OA and those with symptomatic and radiographically confirmed midfoot OA. In contrast to the knee, the relatively vertical orientation of the midfoot joint surfaces may have implications for how load is distributed and potential relationships between BMLs and cartilage loss, which warrant further investigation.

As this study is cross‐sectional, we cannot infer the prognostic significance of BMLs in midfoot OA nor the likelihood that patients with persistent midfoot pain will develop OA. Although diffuse edema in the midfoot bones can be a transient feature of excessive bone stress (31), evidence from other joints such as the knee suggests that BMLs are associated with the subsequent development of cartilage damage and radiographic OA (14, 32, 33, 34). Devices that offload the midfoot and reduce bone stress, such as foot orthoses, appear promising with findings from a pilot randomized controlled trial showing favorable results for pain reduction compared to sham insoles (5). Preliminary findings also show that foot orthoses are able to alter the distribution of BMLs in the midfoot following 12 weeks of treatment for midfoot pain (35). Future research should establish whether the effect of foot orthoses on pain in midfoot OA is mediated through changes in BMLs.

Previous studies using radiography have found the presence of midfoot OA to be associated with foot pain (36) and pain severity (37). At the joint level, Menz et al identified OA in the first and second cuneiform‐metatarsal joints to be associated with prevalent pain, and the number of joints with OA to be moderately associated with foot symptoms (36). In the current study, JSN, cysts, BMLs, and enthesopathy were positively, but weakly associated with symptoms, explaining between 9% and 17% of the variance in pain and 5% of the variance in foot‐related disability in patients with persistent midfoot pain and midfoot OA. Both JSN and osteophytes have shown moderate associations with pain in patients with knee OA (38) and in individual finger joints in hand OA (8, 39). In this study, JSN was the feature most strongly associated with pain severity, but this relationship was strongly attenuated by covariates. In particular, age was the most influential covariate in attenuating the relationship between joint abnormalities and pain. While these findings are consistent with those from other joints showing the limited variance in pain explained by MR structural features, this is the first study to identify and confirm this relationship in midfoot OA.

Studies on knee and hand OA suggest that inflammatory‐mediated abnormalities such as synovitis are important for both incident disease and structural progression (40, 41, 42). In this study, the prevalence of effusion‐synovitis in both patients and controls at the joint‐level in the midfoot was high (45–91%). We were unable to assess for synovial hypertrophy specifically due to the absence of contrast‐enhanced sequences. Other inflammatory features, such as enthesopathy, were associated with greater foot‐related disability likely due to a role of entheses as sites of force dissipation between tendon and bone (43). Although MRI is a highly sensitive modality for identifying tissue pathology, ultrasound is capable of imaging inflammatory features such as synovial hypertrophy and inflammation in midfoot OA (44, 45), which should be explored in future studies.

There are several reasons that may explain the strength of the relationships between structural abnormalities and symptoms. Structural pathology is just one of a multitude of genetic, biological, psychological, contextual, and social factors influencing the pain experience in OA (46). Even in more established research areas such as knee OA, there is conflicting evidence regarding the associations between structural pathology and symptoms (47). An additional challenge in midfoot OA is localizing sites of pain due to the close proximity and small size of the midfoot joints. Traditional approaches using 1 foot per person cannot adequately account for between‐person differences in pain perception, and future studies in foot OA would benefit from within‐person approaches that include both limbs (both symptomatic and asymptomatic) (9, 38). This would also help control for the influence of covariates such as age and BMI, which were identified in this study to be associated with MRI‐detected abnormalities.

The findings of this study should be viewed in the context of certain limitations. First, this was a cross‐sectional study, and relationships between MRI findings and symptoms should be explored longitudinally to investigate causality. The patient sample was also mainly recruited through contact with general practitioners and health clinics, resulting in a sample that was slightly younger, had a lower average BMI, and comprised a greater proportion of female patients compared to studies that have used population‐based sampling strategies (2, 21). As age was positively associated with MRI severity scores, differences between groups may be influenced by age differences as well as clinical presentation/group assignment. As patients with persistent midfoot pain but no radiographic OA were younger, group comparisons and associations between MRI‐abnormalities and symptoms were age adjusted to account for these differences. The identification of differences in MRI features across groups should be viewed in light of the limited sample size and low prevalence and require confirmation in future studies. Secondly, MRI scores have limitations, and despite established intrarater reliability using FOAMRIS (19), the MRI readings were performed by a single radiologist; therefore, consensus readings and a reference atlas could be used in future studies. As mentioned, we did not use contrast enhancement for assessing synovitis or apply novel techniques that may have allowed direct assessment of cartilage thickness; therefore, we recognize we may have underestimated cartilage loss and the severity of synovitis. Finally, as this study used the La Trobe radiographic atlas that includes the medial midfoot joints only, it is possible that OA occurring in the lateral midfoot was not detected, and therefore, MRI abnormalities may be underestimated.

In conclusion, this study found that structural abnormalities of OA not visible on plain‐film radiography, such as BMLs, were associated with persistent midfoot pain in patients without radiographic OA, as well as symptomatic radiographic OA. The location and pattern of BML involvement were consistent with elevated mechanical loading, highlighting the potential role for treatments that reduce midfoot bone stress in the treatment of midfoot pain and OA. Severity scores for JSN, osteophytes, BMLs, cysts, and enthesopathy in the midfoot were not independently associated with pain and foot‐related disability after adjustment for covariates. Longitudinal studies are needed to understand the association of MRI‐detected abnormalities with symptomatic and structural disease progression, and whether changes in MRI abnormalities associate with changes in pain in patients with midfoot OA.

## AUTHOR CONTRIBUTIONS

All authors were involved in drafting the article or revising it critically for important intellectual content, and all authors approved the final version to be submitted for publication. Dr. Arnold had full access to all of the data in the study and takes responsibility for the integrity of the data and the accuracy of the data analysis.

### Study conception and design

Arnold, Halstead, Hill, Redmond.

### Acquisition of data

Arnold, Halstead.

### Analysis and interpretation of data

Arnold, Halstead, Martín‐Hervás, Grainger, Keenan, Hill, Conaghan, McGonagle.

## Supporting information

Disclosure Form

**Supplementary Figure 1** Presence and severity of bone marrow lesions in the navicular, cuneiforms and cuboid, for patients with midfoot osteoarthritis, midfoot pain only and asymptomatic adults. Severity of bone marrow lesion in each bone was scored ranging from 0 to 3, according to the proportion of bone with abnormal signal: 0/Green = None, 1/Yellow = 1%–33%, 2/Orange = 34%–66%, 3/Red = 67%–100%.

**Supplementary Table 1** Prevalence of MRI‐detected joint space narrowing, osteophytes, subchondral cysts and effusion‐synovitis for the midfoot joints in asymptomatic controls, patients with persistent midfoot pain or midfoot osteoarthritis (OA)
**Supplementary Table 2.** Prevalence of MRI‐detected subchondral cysts and effusion‐synovitis for the midfoot joints in asymptomatic controls, patients with persistent midfoot pain or midfoot osteoarthritis (OA)
**Supplementary Table 3.** Prevalence of MRI‐detected bone marrow lesions for the midfoot bones and metatarsal bases in asymptomatic controls, patients with persistent midfoot pain or midfoot osteoarthritis (OA)
**Supplementary Table 4.** Prevalence of MRI‐detected tenosynovitis and enthesopathy in asymptomatic controls, patients with persistent midfoot pain or midfoot osteoarthritis (OA)
**Supplementary Table 5.** Multivariable association between demographics and sum scores for MRI‐detected abnormalities with foot pain and function in patients with persistent midfoot pain and midfoot osteoarthritis

## References

[1] Golightly YM , Gates LS . Foot osteoarthritis: addressing an overlooked global public health problem. Arthritis Care Res (Hoboken) 2021;73:767–9. 32166885 10.1002/acr.24181

[2] Roddy E , Thomas MJ , Marshall M , et al. The population prevalence of symptomatic radiographic foot osteoarthritis in community‐dwelling older adults: cross‐sectional findings from the clinical assessment study of the foot. Ann Rheum Dis 2015;74:156–63. 24255544 10.1136/annrheumdis-2013-203804 PMC4283621

[3] Rathod T , Marshall M , Thomas MJ , et al. Investigations of potential phenotypes of foot osteoarthritis: cross‐sectional analysis from the clinical assessment study of the foot. Arthritis Care Res (Hoboken) 2016;68:217–27. 26238801 10.1002/acr.22677 PMC4819686

[4] Rao S , Baumhauer J , Nawoczenski D . Is barefoot regional plantar loading related to self‐reported foot pain in patients with midfoot osteoarthritis. Osteoarthritis Cartilage 2011;19:1019–25. 21571084 10.1016/j.joca.2011.04.006

[5] Halstead J , Chapman GJ , Gray JC , et al. Foot orthoses in the treatment of symptomatic midfoot osteoarthritis using clinical and biomechanical outcomes: a randomised feasibility study. Clin Rheumatol 2016;35:987–96. 25917211 10.1007/s10067-015-2946-6 PMC4819552

[6] Downes TJ , Chesterton L , Whittle R , et al. Symptomatic course of foot osteoarthritis phenotypes: an 18‐month prospective analysis of community‐dwelling older adults. Arthritis Care Res (Hoboken) 2018;70:1107–12. 29287314 10.1002/acr.23502 PMC6067068

[7] Martel‐Pelletier J , Barr AJ , Cicuttini FM , et al. Osteoarthritis. Nat Rev Dis Primers 2016;2:16072. 27734845 10.1038/nrdp.2016.72

[8] Haugen IK , Bøyesen P , Slatkowsky‐Christensen B , et al. Associations between MRI‐defined synovitis, bone marrow lesions and structural features and measures of pain and physical function in hand osteoarthritis. Ann Rheum Dis 2012;71:899–904. 22121126 10.1136/annrheumdis-2011-200341

[9] Zhang Y , Nevitt M , Niu J , et al. Fluctuation of knee pain and changes in bone marrow lesions, effusions, and synovitis on magnetic resonance imaging. Arthritis Rheum 2011;63:691–9. 21360498 10.1002/art.30148 PMC3056156

[10] Ahedi H , Aitken D , Blizzard L , et al. A population‐based study of the association between hip bone marrow lesions, high cartilage signal, and hip and knee pain. Clin Rheumatol 2014;33:369–76. 24196987 10.1007/s10067-013-2394-0

[11] Vincent TL . Peripheral pain mechanisms in osteoarthritis. Pain 2020;161:S138–46. 33090747 10.1097/j.pain.0000000000001923 PMC7434216

[12] Roemer FW , Demehri S , Omoumi P , et al. State of the art: imaging of osteoarthritis—revisited 2020. Radiology 2020;296:5–21. 32427556 10.1148/radiol.2020192498

[13] Harkey MS , Davis JE , Lu B , et al. Early pre‐radiographic structural pathology precedes the onset of accelerated knee osteoarthritis. BMC Musculoskelet Disord 2019;20:241. 31113401 10.1186/s12891-019-2624-y PMC6530034

[14] Javaid MK , Lynch J , Tolstykh I , et al. Pre‐radiographic MRI findings are associated with onset of knee symptoms: the MOST study. Osteoarthritis Cartilage 2010;18:323–8. 19919856 10.1016/j.joca.2009.11.002 PMC2990960

[15] Chatterton BD , Muller S , Thomas MJ , et al. Inter and intra‐rater repeatability of the scoring of foot pain drawings. J Foot Ankle Res 2013;6;44. 24180324 10.1186/1757-1146-6-44 PMC3831824

[16] Garrow AP , Silman AJ , Macfarlane GJ . The Cheshire Foot Pain and disability survey: a population survey assessing prevalence and associations. Pain 2004;110:378–84. 15275789 10.1016/j.pain.2004.04.019

[17] Menz HB , Munteanu SE , Landorf KB , et al. Radiographic classification of osteoarthritis in commonly affected joints of the foot. Osteoarthritis Cartilage 2007;15:1333–8. 17625925 10.1016/j.joca.2007.05.007

[18] Arnold JB , Halstead J , Grainger AJ , et al. Foot and leg muscle weakness in people with midfoot osteoarthritis. Arthritis Care Res (Hoboken) 2021;73:772–80. 32170831 10.1002/acr.24182

[19] Halstead J , Martín‐Hervás C , Hensor EM , et al. Development and reliability of a preliminary foot osteoarthritis magnetic resonance imaging score. J Rheumatol 2017;44:1257–64. 28572462 10.3899/jrheum.160617 PMC5544115

[20] Garrow AP , Papageorgiou AC , Silman AJ , et al. Development and validation of a questionnaire to assess disabling foot pain. Pain 2000;85:107–13. 10692609 10.1016/s0304-3959(99)00263-8

[21] Thomas MJ , Peat G , Rathod T , et al. The epidemiology of symptomatic midfoot osteoarthritis in community‐dwelling older adults: cross‐sectional findings from the Clinical Assessment Study of the Foot. Arthritis Res Ther 2015;17:178. 26166410 10.1186/s13075-015-0693-3 PMC4499901

[22] Benjamin M , McGonagle D . The anatomical basis for disease localisation in seronegative spondyloarthropathy at entheses and related sites. J Anat 2001;199:503–26. 11760883 10.1046/j.1469-7580.2001.19950503.x PMC1468363

[23] Verhoeven N , Vandeputte G . Midfoot arthritis: diagnosis and treatment. Foot Ankle Surg 2012;18:255–62. 23093120 10.1016/j.fas.2012.04.004

[24] Filippi J , Myerson MS , Scioli MW , et al. Midfoot arthrodesis following multi‐joint stabilization with a novel hybrid plating system. Foot Ankle Int 2012;33:220–5. 22734284 10.3113/FAI.2012.0220

[25] Bowes MA , McLure SW , Wolstenholme CB , et al. Osteoarthritic bone marrow lesions almost exclusively colocate with denuded cartilage: a 3D study using data from the Osteoarthritis Initiative. Ann Rheum Dis 2016;75:1852–7. 26672065 10.1136/annrheumdis-2015-208407

[26] Zubler V , Mengiardi B , Pfirrmann CW , et al. Bone marrow changes on STIR MR images of asymptomatic feet and ankles. Eur Radiol 2007;17:3066–72. 17619194 10.1007/s00330-007-0691-1

[27] Dietrich TJ , da Silva FL , de Abreu MR , et al. First metatarsophalangeal joint‐MRI findings in asymptomatic volunteers. Eur Radiol 2015;25:970–9. 25413967 10.1007/s00330-014-3489-y

[28] Muratovic D , Findlay DM , Cicuttini FM , et al. Bone matrix microdamage and vascular changes characterize bone marrow lesions in the subchondral bone of knee osteoarthritis. Bone 2018;108:193–201. 29331302 10.1016/j.bone.2018.01.012

[29] Felson DT , McLaughlin S , Goggins J , et al. Bone marrow edema and its relation to progression of knee osteoarthritis. Ann Intern Med 2003;139: 330–6. 12965941 10.7326/0003-4819-139-5_part_1-200309020-00008

[30] Lakin RC , DeGnore LT , Pienkowski D . Contact mechanics of normal tarsometatarsal joints. J Bone Joint Surg Am 2001;83:520–8. 11315780 10.2106/00004623-200104000-00006

[31] Schweitzer ME , White LM . Does altered biomechanics cause marrow edema? Radiology 1996;198:851–3. 8628882 10.1148/radiology.198.3.8628882

[32] Felson DT , Niu J , Guermazi A , et al. Correlation of the development of knee pain with enlarging bone marrow lesions on magnetic resonance imaging. Arthritis Care Res (Hoboken) 2007;56:2986–92. 10.1002/art.22851 17763427

[33] Van Oudenaarde K , Jobke B , Oostveen AC , et al. Predictive value of MRI features for development of radiographic osteoarthritis in a cohort of participants with pre‐radiographic knee osteoarthritis: the CHECK study. Rheumatology (Oxford) 2017;56:113–20. 28028160 10.1093/rheumatology/kew368

[34] Sharma L , Nevitt M , Hochberg M , et al. Clinical significance of worsening versus stable preradiographic MRI lesions in a cohort study of persons at higher risk for knee osteoarthritis. Ann Rheum Dis 2016;75:1630–6. 26467570 10.1136/annrheumdis-2015-208129 PMC4833701

[35] Halstead J, Keenan AM, McGonagle D, et al. An exploration into the effect of foot orthoses on bone marrow lesions associated with mechanical foot pain. J Foot Ankle Res 2014;7 Suppl 2:A1. 10.1186/1757-1146-7-S2-A1 PMC4249745 25467741

[36] Menz HB , Munteanu SE , Landorf KB , et al. Radiographic evaluation of foot osteoarthritis: sensitivity of radiographic variables and relationship to symptoms. Osteoarthritis Cartilage 2009;17:298–303. 18789728 10.1016/j.joca.2008.07.011

[37] Arnold JB , Marshall M , Thomas MJ , et al. Midfoot osteoarthritis: potential phenotypes and their associations with demographic, symptomatic and clinical characteristics. Osteoarthritis Cartilage 2019;27:659–66. 30660723 10.1016/j.joca.2018.12.022

[38] Neogi T , Felson D , Niu J , et al. Association between radiographic features of knee osteoarthritis and pain: results from two cohort studies. BMJ 2009;339:b2844. 19700505 10.1136/bmj.b2844 PMC2730438

[39] Kortekaas MC , Kwok WY , Reijnierse M , et al. Osteophytes and joint space narrowing are independently associated with pain in finger joints in hand osteoarthritis. Ann Rheum Dis 2011;70:1835–7. 21742640 10.1136/ard.2010.147553

[40] Haugen IK , Slatkowsky‐Christensen B , Bøyesen P , et al. MRI findings predict radiographic progression and development of erosions in hand osteoarthritis. Ann Rheum Dis 2016;75:117–23. 25204463 10.1136/annrheumdis-2014-205949

[41] Felson DT , Niu J , Neogi T , et al. Synovitis and the risk of knee osteoarthritis: the MOST Study. Osteoarthritis Cartilage 2016;24:458–64. 26432512 10.1016/j.joca.2015.09.013 PMC4761323

[42] De Lange‐Brokaar BJ , Ioan‐Facsinay A , Yusuf E , et al. Evolution of synovitis in osteoarthritic knees and its association with clinical features. Osteoarthritis Cartilage 2016;24:1867–74. 27262546 10.1016/j.joca.2016.05.021

[43] Shaw HM , Benjamin M . Structure–function relationships of entheses in relation to mechanical load and exercise. Scand J Med Sci Sports 2007;17:303–15. 17490450 10.1111/j.1600-0838.2007.00689.x

[44] Camerer M , Ehrenstein B , Hoffstetter P , et al. High‐resolution ultrasound of the midfoot: sonography is more sensitive than conventional radiography in detection of osteophytes and erosions in inflammatory and non‐inflammatory joint disease. Clin Rheumatol 2017;36:2145–9. 28478580 10.1007/s10067-017-3658-x

[45] Zabotti A , Filippou G , Canzoni M , et al. OMERACT agreement and reliability study of ultrasonographic elementary lesions in osteoarthritis of the foot. RMD Open 2019;5:e000795. 30997148 10.1136/rmdopen-2018-000795 PMC6443136

[46] Neogi T. The epidemiology and impact of pain in osteoarthritis. Osteoarthritis Cartilage 2013;21:1145–53. 23973124 10.1016/j.joca.2013.03.018 PMC3753584

[47] Yusuf E , Kortekaas MC , Watt I , et al. Do knee abnormalities visualised on MRI explain knee pain in knee osteoarthritis? A systematic review. Ann Rheum Dis 2011;70:60–7. 20829200 10.1136/ard.2010.131904

