# Supplementary material for: Bone Marrow Lesions and Magnetic Resonance Imaging–Detected Structural Abnormalities in Patients With Midfoot Pain and Osteoarthritis: A Cross‐Sectional Study
Source: Arthritis Care Res (Hoboken). 2022 Dec 2;75(5):1113–22. doi: 10.1002/acr.24955 (PMC10952448; doi:10.1002/acr.24955)
Supplement: Supplementary file 2 — Supplementary Figure 1 Presence and severity of bone marrow lesions in the navicular, cuneiforms and cuboid, for patients with midfoot osteoarthritis, midfoot pain only and asymptomatic adults. Severity of bone marrow lesion in each bone was scored ranging from 0 to 3, according to the proportion of bone with abnormal signal: 0/Green = None, 1/Yellow = 1%–33%, 2/Orange = 34%–66%, 3/Red = 67%–100%. [file ACR-75-1113-s001.docx]

**Supplementary Figure 1**


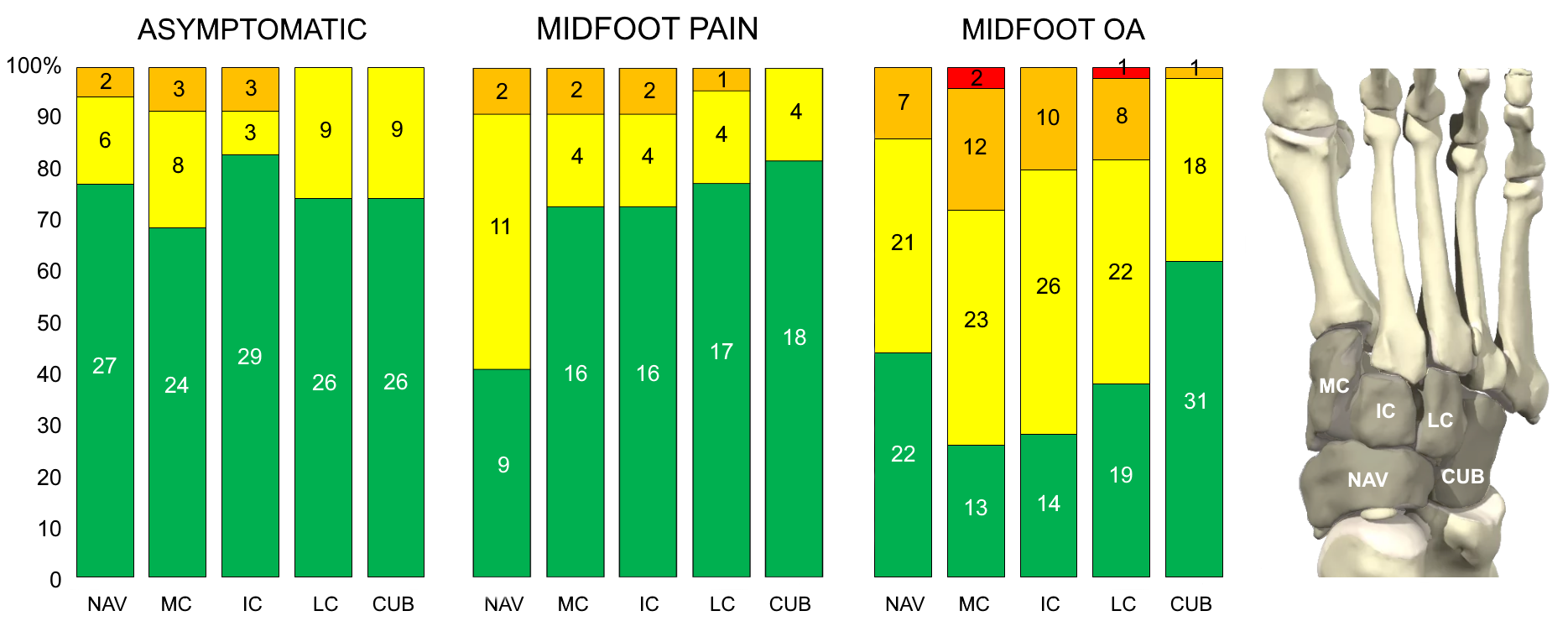


**Supplementary Figure 1.** Presence and severity of bone marrow lesions in the navicular, cuneiforms and cuboid, for patients with midfoot osteoarthritis, midfoot pain only and asymptomatic adults. Severity of bone marrow lesion in each bone was scored ranging from 0 to 3, according to the proportion of bone with abnormal signal: 0/Green = None, 1/Yellow = 1%–33%, 2/Orange = 34%–66%, 3/Red = 67%–100%.
