# Supplementary material for: Bone Marrow Lesions and Magnetic Resonance Imaging–Detected Structural Abnormalities in Patients With Midfoot Pain and Osteoarthritis: A Cross‐Sectional Study
Source: Arthritis Care Res (Hoboken). 2022 Dec 2;75(5):1113–22. doi: 10.1002/acr.24955 (PMC10952448; doi:10.1002/acr.24955)
Supplement: Supplementary file 3 — Supplementary Table 1 Prevalence of MRI‐detected joint space narrowing, osteophytes, subchondral cysts and effusion‐synovitis for the midfoot joints in asymptomatic controls, patients with persistent midfoot pain or midfoot osteoarthritis (OA) Supplementary Table 2. Prevalence of MRI‐detected subchondral cysts and effusion‐synovitis for the midfoot joints in asymptomatic controls, patients with persistent midfoot pain or midfoot osteoarthritis (OA) Supplementary Table 3. Prevalence of MRI‐detected bone marrow lesions for the midfoot bones and metatarsal bases in asymptomatic controls, patients with persistent midfoot pain or midfoot osteoarthritis (OA) Supplementary Table 4. Prevalence of MRI‐detected tenosynovitis and enthesopathy in asymptomatic controls, patients with persistent midfoot pain or midfoot osteoarthritis (OA) Supplementary Table 5. Multivariable association between demographics and sum scores for MRI‐detected abnormalities with foot pain and function in patients with persistent midfoot pain and midfoot osteoarthritis [file ACR-75-1113-s002.docx]

**Supplementary Table 1.** Prevalence of MRI-detected joint space narrowing, osteophytes, subchondral cysts and effusion-synovitis for the midfoot joints in asymptomatic controls, patients with persistent midfoot pain or midfoot osteoarthritis (OA)

|  | **Joint space narrowing (≥2)** | | | | **Osteophytes (≥2)** | | | | |
| --- | --- | --- | --- | --- | --- | --- | --- | --- | --- |
| **Joint** | **Control** | **Midfoot**  **pain** | **Midfoot**  **OA** | **p-value** | **Control** | **Midfoot**  **pain** | **Midfoot**  **OA** | **p-value** |  |
| **Talonavicular** (*n*, %) | 2 (6) | 0 (0) | 3 (6) | 0.506 | 2 (6) | 5 (23) | 6 (12) | 0.160 |  |
| **Navicular-medial cuneiform** (*n*, %) | 2 (6) | 0 (0) | 3 (6) | 0.506 | 2 (6) | 1 (5) | 6 (12) | 0.451 |  |
| **Navicular-intermediate cuneiform** (*n*, %) | 2 (6) | 0 (0) | 4 (8) | 0.397 | 0 (0) | 0 (0) | 2 (4) | 0.313 |  |
| **Navicular-lateral cuneiform** (*n*, %) | 1 (3) | 0 (0) | 4 (8) | 0.275 | 1 (3) | 0 (0) | 2 (4) | 0.631 |  |
| **Calcaneus-cuboid** (*n*, %) | 3 (9) | 0 (0) | 2 (4) | 0.313 | 1 (3) | 3 (14) | 2 (4) | 0.181 |  |
| **Cuneiform-first metatarsal** (*n*, %) | 2 (6) | 1 (5) | 9 (18) | 0.113 | 1 (3) | 2 (9) | 7 (14) | 0.221 |  |
| **Cuneiform-second metatarsal** (*n*, %) | 2 (6) | 3 (14) | 16 (32) | 0.008* | 1 (3) | 5 (23) | 9 (18) | 0.059 |  |
| **Cuneiform-third metatarsal** (*n*, %) | 2 (6) | 0 (0) | 12 (24) | 0.006* | 2 (6) | 1 (5) | 8 (16) | 0.175 |  |
| **Cuboid-fourth metatarsal** (*n*, %) | 1 (3) | 1 (5) | 4 (8) | 0.580 | 0 (0) | 0 (0) | 6 (12) | 0.027* |  |
| **Cuboid-fifth metatarsal** (*n*, %) | 0 (0) | 0 (0) | 3 (6) | 0.172 | 0 (0) | 0 (0) | 1 (2) | 0.562 |  |

* statistically significant difference between control, midfoot pain and midfoot OA groups (p<0.05)

**Supplementary Table 2.** Prevalence of MRI-detected subchondral cysts and effusion-synovitis for the midfoot joints in asymptomatic controls, patients with persistent midfoot pain or midfoot osteoarthritis (OA)

|  | **Subchondral cyst (0,1)** | | | | **Effusion-Synovitis (0,1)** | | | | |
| --- | --- | --- | --- | --- | --- | --- | --- | --- | --- |
| **Joint** | **Control** | **Midfoot**  **pain** | **Midfoot**  **OA** | **p-value** | **Control** | **Midfoot**  **pain** | **Midfoot**  **OA** | **p-value** |  |
| **Talonavicular** (*n*, %) | 4 (11) | 3 (14) | 6 (12) | 0.969 | 28 (80) | 18 (82) | 37 (74) | 0.700 |  |
| **Navicular-medial cuneiform** (*n*, %) | 6 (17) | 5 (23) | 14 (28) | 0.506 | 21 (60) | 16 (73) | 29 (58) | 0.481 |  |
| **Navicular-intermediate cuneiform** (*n*, %) | 4 (11) | 0 (0) | 11 (22) | 0.040* | 18 (51) | 10 (45) | 24 (48) | 0.902 |  |
| **Navicular-lateral cuneiform** (*n*, %) | 2 (6) | 1 (9) | 6 (12) | 0.451 | 20 (57) | 11 (50) | 22 (44) | 0.490 |  |
| **Calcaneus-cuboid** (*n*, %) | 5 (14) | 0 (0) | 5 (10) | 0.192 | 21 (60) | 16 (73) | 34 (68) | 0.579 |  |
| **Cuneiform-first metatarsal** (*n*, %) | 6 (17) | 4 (18) | 17 (34) | 0.147 | 30 (86) | 20 (91) | 44 (88) | 0.933 |  |
| **Cuneiform-second metatarsal** (*n*, %) | 5 (14) | 3 (14) | 13 (26) | 0.298 | 30 (86) | 17 (77) | 39 (78) | 0.680 |  |
| **Cuneiform-third metatarsal** (*n*, %) | 6 (17) | 1 (5) | 20 (40) | 0.002* | 28 (80) | 16 (73) | 40 (80) | 0.760 |  |
| **Cuboid-fourth metatarsal** (*n*, %) | 4 (11) | 0 (0) | 9 (18) | 0.097 | 27 (77) | 17 (77) | 42 (84) | 0.676 |  |
| **Cuboid-fifth metatarsal** (*n*, %) | 0 (0) | 0 (0) | 4 (8) | 0.094 | 30 (86) | 18 (82) | 38 (76) | 0.530 |  |

* statistically significant difference between control, midfoot pain and midfoot OA groups (p<0.05)

**Supplementary Table 3.** Prevalence of MRI-detected bone marrow lesions for the midfoot bones and metatarsal bases in asymptomatic controls, patients with persistent midfoot pain or midfoot osteoarthritis (OA)

|  | **Bone marrow lesion (≥2)** | | | |
| --- | --- | --- | --- | --- |
| **Bone** | **Control** | **Midfoot**  **pain** | **Midfoot**  **OA** | **p-value** |
| **Calcaneus** (*n*, %) | 0 (0) | 0 (0) | 3 (6) | 0.172 |
| **Talus** (*n*, %) | 0 (0) | 1 (5) | 3 (6) | 0.348 |
| **Navicular** (*n*, %) | 2 (6) | 2 (9) | 7 (14) | 0.455 |
| **Medial cuneiform** (*n*, %) | 3 (9) | 2 (9) | 14 (28) | 0.034* |
| **Intermediate cuneiform** (*n*, %) | 3 (9) | 2 (9) | 10 (20) | 0.248 |
| **Lateral cuneiform** (*n*, %) | 0 (0) | 1 (5) | 9 (18) | 0.013* |
| **Cuboid** (*n*, %) | 0 (0) | 0 (0) | 1 (2) | 0.562 |
| **Metatarsal base 1** (*n*, %) | 0 (0) | 0 (0) | 4 (8) | 0.094 |
| **Metatarsal base 2** (*n*, %) | 1 (3) | 2 (9) | 14 (28) | 0.005* |
| **Metatarsal base 3** (*n*, %) | 0 (0) | 1 (5) | 9 (18) | 0.013* |
| **Metatarsal base 4** (*n*, %) | 0 (0) | 0 (0) | 5 (10) | 0.050 |
| **Metatarsal base 5** (*n*, %) | 0 (0) | 0 (0) | 0 (0) | N/A |

* statistically significant difference between control, midfoot pain and midfoot OA groups (p<0.05)

**Supplementary Table 4.** Prevalence of MRI-detected tenosynovitis and enthesopathy in asymptomatic controls, patients with persistent midfoot pain or midfoot osteoarthritis (OA)

|  | | **Enthesopathy (0,1)** | | | | |  | | **Tenosynovitis (≥2)** | | | | |
| --- | --- | --- | --- | --- | --- | --- | --- | --- | --- | --- | --- | --- | --- |
| **Enthesis** | **Control** | | **Midfoot**  **pain** | **Midfoot**  **OA** | **p-value** | **Tendon** | | **Control** | | **Midfoot**  **pain** | **Midfoot**  **OA** | **p-value** |  |
| **Tibialis anterior-first metatarsal** (*n*, %) | 0 (0) | | 0 (0) | 0 (0) | N/A | **Tibialis anterior** (*n*, %) | | 0 (0) | | 0 (0) | 0 (0) | N/A |  |
| **Tibialis anterior-medial cuneiform** (*n*, %) | 0 (0) | | 0 (0) | 1 (2) | 0.562 | **Extensor hallucis longus** (*n*, %) | | 0 (0) | | 0 (0) | 0 (0) | N/A |  |
| **Tibialis posterior-navicular** (*n*, %) | 0 (0) | | 2 (9) | 2 (4) | 0.210 | **Extensor digitorum longus** (*n*, %) | | 0 (0) | | 0 (0) | 1 (2) | 0.677 |  |
| **Tibialis posterior-medial cuneiform** (*n*, %) | 0 (0) | | 2 (9) | 4 (8) | 0.210 | **Fibularis longus** (*n*, %) | | 6 (17) | | 0 (0) | 8 (16) | 0.019* |  |
| **Tibialis posterior-lateral cuneiform** (*n*, %) | 0 (0) | | 1 (5) | 0 (0) | 0.142 | **Fibularis brevis** (*n*, %) | | 0 (0) | | 0 (0) | 1 (2) | 0.471 |  |
| **Tibialis posterior-metatarsal two/three/four** (*n*, %) | 1 (3) | | 0 (0) | 1 (2) | 0.737 | **Tibialis posterior** (*n*, %) | | 2 (6) | | 7 (32) | 8 (16) | 0.063 |  |
| **Fibularis longus-medial cuneiform** (*n*, %) | 6 (17) | | 0 (0) | 5 (10) | 0.116 | **Flexor digitorum longus** (*n*, %) | | 0 (0) | | 1 (5) | 1 (2) | 0.193 |  |
| **Fibularis longus-first metatarsal** (*n*, %) | 8 (23) | | 6 (27) | 7 (14) | 0.359 | **Flexor hallucis longus** (*n*, %) | | 2 (6) | | 2 (9) | 1 (2) | 0.250 |  |
| **Fibularis brevis-fifth metatarsal** (*n*, %) | 0 (0) | | 0 (0) | 1 (2) | 0.562 |  | |  | |  |  |  |  |
| **Tibialis posterior-calcaneus enthesis groove** (*n*, %) | 0 (0) | | 1 (5) | 2 (4) | 0.468 |  | |  | |  |  |  |  |
| **Fibularis longus-calcaneus enthesis groove** (*n*, %) | 8 (23) | | 3 (14) | 6 (12) | 0.383 |  | |  | |  |  |  |  |
| **Tibialis posterior-navicular enthesis groove** (*n*, %) | 0 (0) | | 7 (32) | 4 (8) | <0.001* |  | |  | |  |  |  |  |
| **Tibialis anterior-medial cuneiform enthesis groove** (*n*, %) | 0 (0) | | 0 (0) | 1 (2) | 0.562 |  | |  | |  |  |  |  |
| **Fibularis longus-cuboid enthesis groove** (*n*, %) | 6 (17) | | 4 (18) | 11 (22) | 0.842 |  | |  | |  |  |  |  |

TP: Tibilis posterior, FL: Fibularis longus, TA: Tibialis anterior

* statistically significant difference between control, midfoot pain and midfoot OA groups (p<0.05)

**Supplementary Table 5.** Multivariable association between demographics and sum scores for MRI-detected abnormalities with foot pain and function in patients with persistent midfoot pain and midfoot osteoarthritis

|  | **MFPDI Pain** | | **MFPDI Function** | |
| --- | --- | --- | --- | --- |
|  | **B (95% CI)** | **p-value** | **B (95% CI)** | **p-value** |
| Age | 0.020 (-0.016, 0.057) | 0.264 | -0.004 (-0.048, 0.040) | 0.858 |
| Gender | 0.471 (-0.533, 1.474) | 0.353 | 0.685 (-0.861, 2.232) | 0.379 |
| Body mass index | -0.002 (-0.099, 0.095) | 0.966 | 0.130 (-0.015, 0.275) | 0.079 |
| Joint space narrowing | 0.190 (-0.038, 0.418) | 0.100 | - | - |
| Bone marrow lesions | -0.009 (-0.176, 0.158) | 0.914 | - | - |
| Cysts | -0.020 (-0.413, 0.373) | 0.920 | - | - |
| Enthesopathy | - | - | 0.359 (-0.319, 1.037) | 0.295 |

Data are presented as unstandardised B coefficients with 95% CIs from multivariable models

MFPDI: Manchester foot pain disability index
